# Preoperative Quality of Life and Mental Health Can Predict Postoperative Outcomes and Quality of Life after Colorectal Cancer Surgery

**DOI:** 10.3390/medicina59061129

**Published:** 2023-06-12

**Authors:** Jia-Hao Law, Jerrald Lau, Ning-Qi Pang, Athena Ming-Gui Khoo, Wai-Kit Cheong, Bettina Lieske, Choon-Seng Chong, Kuok-Chung Lee, Ian Jse-Wei Tan, Bei-En Siew, Yi-Xuan Lim, Chermaine Ang, Lina Choe, Wei-Ling Koh, Alyssa Ng, Ker-Kan Tan

**Affiliations:** 1 Department of Surgery, National University Hospital, Singapore 119074, Singapore; law.jiahao@gmail.com (J.-H.L.);; 2 Department of Surgery, Yong Loo Lin School of Medicine, National University of Singapore, Singapore 119077, Singapore; 3 Saw Swee Hock School of Public Health, National University of Singapore, Singapore 119077, Singapore

**Keywords:** colorectal cancer, colorectal cancer outcomes, health-related quality of life, mental health

## Abstract

*Background and Objectives*: It remains unclear which domains of preoperative health-related quality of life (HRQOL) and mental health are predictive of postoperative clinical and patient-reported outcomes in colorectal cancer (CRC) patients. *Materials and Methods:* A prospective cohort of 78 CRC patients undergoing elective curative surgery was recruited. The EORTC QLQ-C30 and HADS questionnaires were administered preoperatively and one month after surgery. *Results:* Preoperative cognitive functioning scores (95% CI 0.131–1.158, *p* = 0.015) and low anterior resection (95% CI 14.861–63.260, *p* = 0.002) independently predicted poorer 1-month postoperative global QOL. When postoperative complications were represented using the comprehensive complication index (CCI), poorer preoperative physical function scores were associated with higher CCI scores (B = −0.277, *p* = 0.014). Preoperative social function score (OR = 0.925, 95% CI 0.87 to 0.99; *p* = 0.019) was an independent predictor for 30-day readmission, while physical functioning score (OR = −0.620, 95% CI −1.073–−0.167, *p* = 0.008) was inversely related to the length of hospitalization. The overall regressions for 1-month postoperative global QOL (R^2^: 0.546, F: 1.961, *p* = 0.023) and 30-day readmission (R^2^: 0.322, χ2: 13.129, *p* < 0.001) were statistically significant. *Conclusions:* Various QLQ-C30 domains were found to be predictive of postoperative outcomes, including complications, readmission, and length of hospitalization. Preoperative cognitive dysfunction and low AR were independent predictors of poorer postoperative global QOL. Future research should seek to examine the efficacy of targeting specific baseline QOL domains in improving clinical as well as patient-reported outcomes after CRC surgery.

## 1. Introduction

The evaluation of health-related quality of life (HRQOL) in colorectal cancer (CRC) survivors allows for a better understanding of the patient’s experience of the disease, treatment, and recovery journey aside from just oncological outcomes, which have improved considerably over the decades [1,2]. More importantly, it provides insight into risk factors for low postoperative HRQOL, which can guide the implementation of appropriate interventions and survivorship care plans to minimize the impact on future patients [3].

The assessment of baseline preoperative HRQOL has been found to provide prognostic information on survival in CRC patients [4] as well as postoperative mortality in CRC patients who undergo surgery [5]. Alexandros et al. recently highlighted the use of the European Organization for Research and Treatment of Cancer (EORTC) QLQ-C30 as an accurate tool in predicting postoperative complications after CRC surgery [6]. The EORTC QLQ-C30 is an internationally validated tool for the measurement of patient-reported HRQOL in cancer patients [7,8]. However, it remains unclear which domains of preoperative HRQOL are predictive of post-surgical outcomes and the relationship between pre- and postoperative HRQOL.

On the other hand, psychological factors have been observed to influence CRC patients’ HRQOL. Anxiety and depression are known to be two of the most prevalent mental health conditions among cancer patients, with rates ranging from 13–40% [9,10]. Studies involving the widely used and validated Hospital Anxiety and Depression Scale (HADS) have found that both the anxiety and depression subscales of the HADS were correlated with poorer general QOL and emotional functioning scores, but that depression scores tended to have stronger associations [11]. Nonetheless, there is scant literature examining whether preoperative HADS scores might be associated with postoperative clinical or patient-reported outcomes.

Hypothetically, preoperative QOL targets could potentially be optimized to improve clinical as well as patient-reported outcomes after CRC surgery, should a relationship between baseline preoperative HRQOL and psychological health with postoperative outcomes and postoperative HRQOL exist. Therefore, the aim of this exploratory study was to examine if the various domains of preoperative HRQOL (measured using the EORTC QLQ-C30) as well as depression and anxiety (measured using the HADS) can significantly predict postoperative clinical and patient-reported outcomes in a prospective cohort of CRC patients who underwent curative CRC surgery.

## 2. Methods

### 2.1. Sample and Setting

A prospective cohort of 78 newly diagnosed CRC patients who underwent elective curative surgery agreed to participate in the study and was recruited from the outpatient clinic of the National University Hospital between February 2018 and August 2021. We excluded patients whose primary cancer was of other origin, patients who underwent endoscopic resection of cancer, emergency surgeries, and those who were unable to provide informed consent. There were no patients with pre-existing psychological disorders in the study cohort. Patients who were assessed to be at risk of anxiety or depression from the Hospital Anxiety and Depression (HADS) questionnaire were not excluded as long as they were assessed to be fit to give consent for the study. Anterior resection and low anterior resection were defined as oncological resection of the tumor with colorectal anastomosis created above and below the peritoneal reflection, respectively. Patients with rectal cancer who underwent neoadjuvant radiotherapy either received short-course (25 Gy in 5 fractions) or long-course (45–50.4 Gy in 25–28 fractions) radiotherapy based on recommendations from a multidisciplinary tumor board, which consists of oncologists, radiologists, radiation oncologists, and surgeons. This study was approved by the Institutional Review Board of National Healthcare Group (NHG DSRB ref: 2017/00518, approval date 23 June 2017).

### 2.2. Measures

#### 2.2.1. Sociodemographic and Clinical Data

Sociodemographic and clinical data were retrieved from the medical records of participant patients using a standardized data collection form (DCF). Data on patients’ gender, tumor site, date of operation, type of operation, tumor staging on diagnosis, postoperative complications, return to the operating room and/or readmission within 30 days, intraoperative blood loss, preoperative comorbidities, and length of stay post-operation were retrieved from the institution’s electronic medical records system.

#### 2.2.2. European Organization for Research and Treatment of Cancer Quality of Life Questionnaire (EORTC QLQ-C30)

The EORTC QLQ-C30 is a 30-item internationally validated questionnaire measuring patient-reported QOL within the context of cancer and oncology. The QLQ-C30 comprises a global health status (QOL) scale, five functional scales (physical, role, emotional, cognitive, and social functioning), three symptom scales (fatigue, nausea/vomiting, and pain), and six individual items (dyspnea, insomnia, appetite loss, constipation, diarrhea, and financial difficulties) [12].

#### 2.2.3. Hospital Anxiety and Depression Scales (HADS)

The HADS is a 14-item, internationally validated questionnaire measuring patient-reported mental health in the clinical and hospital settings. The HADS contains two subscales—anxiety (7 items) and depression (7 items)—which are composited separately to provide an anxiety and depression scores, respectively [13]. Higher scores denote a more severe degree of perceived anxiety or depression.

#### 2.2.4. Comprehensive Complication Index (CCI)

Postoperative complications were represented using the CCI, which integrates all complications of the Clavien-Dindo classification (CDC) and offers a metric approach to measuring morbidity [14]. It is a more precise grading system to measure complications compared to the conventional CDC, as it accounts for the severity and total number of postoperative complications [15].

### 2.3. Procedure

After obtaining informed consent and ascertaining eligibility, participants were administered the EORTC QLQ-C30 and HADS at two timepoints by the study team—the preoperative consultation (i.e., baseline) and the follow-up consultation one month after their elective curative surgery (i.e., postoperative). The questionnaire was administered prior to stoma reversal for all patients who had stoma creation during their index surgery. 

### 2.4. Statistical Analyses

All patient data was entered into the institution’s secured research database system (REDCap) by one study team member and independently cross-validated by a second member of the study team. All patient identifiers were removed before the data was extracted and analyzed via IBM SPSS Statistics software Version 25 (Chicago, IL, USA). 

Descriptive analyses were used to present the sample’s key demographics (e.g., age, gender), baseline disease and clinical factors (e.g., cancer staging, site of tumor, type of surgery), and surgical outcomes (e.g., postoperative morbidity, readmission). Univariate tests of association between exposures, potential confounders, and outcomes of interest were conducted using linear regression for continuous outcomes (i.e., postoperative global QOL score, CCI score, length of stay post-operation). Univariate binary logistic regression was used for categorical binary outcomes (i.e., incidence of postoperative complications, readmission within 30 days of operation). Potential confounders that were statistically significant in univariate analyses were included in the respective multivariable linear or logistic regression models. A *p*-value of <0.05 was defined as statistical significance for all tests of association. 

## 3. Results

Rectal cancer was diagnosed in 29 (37.2%) patients, and 61 (78.2%) patients underwent minimally invasive surgery. There were four (5.1%) patients with significant postoperative complications, which were Clavien-Dindo Grade III. There was no 30-day postoperative mortality. An overview of the disease factors and surgical outcomes can be found in Table 1. 

Higher preoperative emotional function (*p* = 0.010), cognitive function (*p* = 0.010), and global QOL (*p* = 0.037) scores were associated with higher 1-month postoperative global QOL. Conversely, anxiety (*p* = 0.037) and depression (*p* = 0.010) were associated with poorer 1-month postoperative global QOL. After adjusting for various patient, disease, and surgical factors, only preoperative cognitive functioning scores (95% CI 0.131–1.158, *p* = 0.015) and low anterior resection (95% CI 14.861–63.260, *p* = 0.002) independently predicted poorer 1-month postoperative global QOL, as illustrated in Table 2. The overall regression model was statistically significant (R^2^: 0.546, F: 1.961, *p* = 0.023). Preoperative health, represented by ASA score, neoadjuvant treatment, disease stage, postoperative complications, and readmissions, were not associated with poorer 1-month postoperative global QOL.

Patients who developed postoperative complications had lower preoperative global QOL scores (mean score of 64.46 vs. 72.16; *p* = 0.081), but this was not statistically significant. No significant association was found between the various domain scores and postoperative morbidity (Table 3). When postoperative complications were represented using CCI, poorer preoperative physical function scores were associated with higher CCI scores (B = −0.277, *p* = 0.014), though the overall regression model was not statistically significant (R^2^: 0.134, F: 1.320, *p* = 0.249). 

Lower preoperative cognitive score (mean score of 72.92 vs. 91.43; *p* = 0.005) and social (mean score of 72.92 vs. 91.43; *p* = 0.004), functioning scores were associated with 30-day readmission. Preoperative social function score (OR = 0.925, 95% CI 0.87 to 0.99; *p* = 0.019), in particular, was found to be an independent predictor for 30-day readmission. The overall regression model for 30-day readmission (R^2^: 0.322, χ^2^: 13.129, *p* < 0.001) was statistically significant. On the other hand, the preoperative physical functioning score (OR = −0.620, 95% CI −1.073–−0.167, *p* = 0.008) was inversely related to the length of hospitalization, though the overall regression model was not statistically significant (R^2^: 0.156, F: 1.577, *p* = 0.148).

## 4. Discussion

Patient-reported outcome measures (PROMs) capture a patient’s perception of their health status through questionnaires by allowing them to report on their quality of life, daily functioning, symptoms, and mental health. As cancer survivorship continues to improve, there is increasing emphasis on the assessment of HRQOL as a patient-reported outcome, as post-treatment HRQOL becomes an important yardstick of treatment success. The EORTC QLQ-C30 is one of the most widely used HRQOL questionnaires in cancer research and assesses important functioning domains and common cancer symptoms (e.g., fatigue, pain, nausea, and appetite loss), while the HADS focuses on psychological distress in cancer patients. When used concurrently, they give a comprehensive insight into the patient’s perceived health status during their cancer journey. 

The QLQ-C30 “global QOL” domain score reflects the patients’ perception of overall health and overall quality of life. Consistent with existing literature, despite being sphincter-preserving, low anterior resection (AR) was found to be an independent predictor of poorer postoperative global QOL [16]. This was reported to stem from a postoperative increase in defecation-related problems, in particular with continence and controlling their stools [17]. Our results revealed that patients with low anastomosis have significantly poorer global QOL than those with high AR. This was shown to be even more important than the anxiety and depression related to cancer surgery, postoperative complications, and readmissions in influencing postoperative global QOL scores. Significant improvements in neoadjuvant treatment in the past decades, coupled with progress in the development of novel surgical techniques for low rectal cancer, have made sphincter-preserving surgery more commonly performed in recent years. Our results warrant further evaluation of impaired QOL after low AR, even with modern treatment approaches. The goal should be to determine how global QOL was impaired by surgery so that patients can be better selected for sphincter-preserving surgery without compromising postoperative QOL. 

The “cognitive function” domain in QLQ-C30 is a composite score derived from the patient’s perception of memory and concentration abilities. Preoperative cognitive dysfunction can lead to increased dependency in daily life, and such dependency can negatively affect the overall QOL [18,19]. Pan et al. reported that this association was more clearly evident for severe cognitive dysfunction [20]. The results from our study showed lower preoperative cognitive function scores to be an independent predictor of poorer postoperative global QOL scores regardless of disease stage, type of surgery, and surgical outcomes. This highlights the importance of further assessing patients with reported low preoperative cognitive function scores, so that appropriate resources can be provided to the patient and family to optimize their postoperative QOL. 

The role of the preoperative QLQ-C30 score in predicting postoperative complications in patients undergoing curative surgery for CRC remains unclear. Although Alexandros et al. [6] suggested that it was accurate in predicting postoperative complications, it was unclear which domain score was used in the analysis. In our study, although poorer physical functioning scores were associated with higher CCI scores, they failed to predict CCI after accounting for other domain scores as potential confounders. Interestingly, the preoperative social function score, which takes into account the impact of CRC treatment and diagnosis on the patient’s family and social life, was found to be an independent predictor of postoperative readmission. Poorer social function scores may reflect the patient’s suboptimal social support or a perception of social and family life being disrupted by the diagnosis of CRC. This may translate to inferior postoperative care and recovery when the patient is discharged home, thus accounting for higher readmission rates. Assessment of the patient’s social support could perhaps be conducted in greater detail preoperatively so that appropriate assistance can be rendered to facilitate better discharge planning and thereby reduce readmission.

Higher preoperative physical functioning scores were also found to correlate with a shorter length of hospitalization. “Physical function” domain score reflects the patient’s physical fitness and encompasses the patient’s perceived ability to perform various physical activities ranging from activities of daily living (ADLs) to strenuous activities such as taking long walks and carrying heavy shopping bags. Baseline physical fitness contributed to a shorter duration of hospitalization as patients would not only be able to better tolerate surgical injury and stress but would also likely be able to ambulate sooner after surgery and participate in postoperative rehabilitation. Although this may appear intuitive, “prehabilitation” is still not routine for patients undergoing major CRC surgery. Prehabilitation refers to the preoperative optimization of patients’ nutritional status and physical fitness in the weeks leading up to surgery [21]. It has been shown to reduce postoperative complications and the length of stay after major surgery [22]. Emphasis should therefore be placed on refining prehabilitation programs to further improve surgical outcomes. 

Mental well-being is a key component of HRQOL. Both depression and anxiety were found to be associated with poorer postoperative global QOL in our study, though they were not independent predictors on multivariate analysis. Psychological distress has been shown to negatively impact surgery outcomes [23] and was a significant predictor of length of stay [24], readmission [25], and morbidity [26] in various studies. Despite available treatment strategies for mood disorders, mental health optimization is not commonly addressed before surgery. There should be greater emphasis on the perioperative emotional and psychological support of patients undergoing major cancer surgery, with the aim of improving postoperative outcomes as well as quality of life. A psychologist and psychiatrist should review the patient prior to surgery and commence counseling and treatment, and follow-up should be continued after surgery. Although there may be limited time for mental health to be optimized prior to surgery, especially in patients who are not undergoing neoadjuvant therapy, early identification of patients at risk of depression and anxiety, coupled with prompt intervention, will help to alleviate associated postoperative morbidity and delays in recovery following surgery. 

The main limitation of this study lies in its relatively small sample size. Thus, Type II errors, which may potentially understate the purported influence of preoperative HRQOL on postoperative outcomes, are inevitable. There is also a larger proportion of rectal cancers in our sample compared to existing literature, which reports rectal cancer to constitute 25–30% of all CRCs [27]. This overrepresentation may possibly influence the results of our study. Nonetheless, our study team is concurrently recruiting patients from other surgical institutions in the nation to expand our study population and validate our results. QOL scores as well as CRC-specific EORTC QLQ-C29 scores are being collected at various postoperative timepoints to allow us to explore correlations between QOL and short- and long-term outcomes. Moving ahead, other QOL scales that measure social and cognitive function in greater depth could also be utilized to better examine how they influence postoperative outcomes, as the EORTC QLQ-C30 only has two items each on social and cognitive functioning that make up the domain scores.

## 5. Conclusions

In summary, various QLQ-C30 domains were found to be predictive of postoperative outcomes, including postoperative complications, 30-day readmission, and length of hospitalization. Preoperative cognitive dysfunction and low AR were also found to be independent predictors of poorer postoperative global QOL. Our findings can serve as a springboard for future studies to examine the efficacy of targeting specific baseline QOL domains in improving clinical as well as patient-reported outcomes after CRC surgery. 

## Figures and Tables

**Table 1:** Key demographics, disease factors, and surgical outcomes.

Profile	(% or Range)
	*N* = 78
**Median age (years)**	66 (45–89)
**Gender**	
Male	44 (56.4)
Female	33 (43.6)
**American Society of Anesthesiologists (ASA) class**	
I	6 (7.7)
II	51 (65.4)
III	21 (26.9)
**Stage**	
I	15 (19.2)
II	24 (30.8)
III	34 (43.6)
IV	5 (6.4)
**Site of tumor**	
Colon	49 (62.8)
Rectal	29 (37.2)
**Surgical approach**	
Minimally invasive	61 (78.2)
Open	17 (21.8)
**Stoma**	27 (34.6)
Ileostomy	18 (66.7)
Colostomy	9 (33.3)
**Surgery**	
Segmental (Left/Right)	18 (23.1)
Subtotal/total colectomy	4 (5.1)
Anterior resection	38 (48.7)
Low anterior resection	10 (12.8)
Hartmanns surgery	2 (2.6)
Abdominoperineal resection	6 (7.7)
**Neoadjuvant treatment**	
Chemotherapy	9 (11.5)
Radiotherapy	13 (16.7)
**Morbidity**	34 (43.6)
Clavien I	17 (21.8)
Clavien II	13 (16.7)
Clavien III	4 (5.1)
**30-day readmission**	8 (10.3)

**Table 2:** Univariate and multivariate analyses for predictors of one month postoperative global QOL; Univariate analysis: Multivariate analysis (R^2^: 0.546, F: 1.961, *p* = 0.023).

	Univariate Analysis	Multivariate Analysis (R^2^: 0.546, F: 1.961, *p* = 0.023).		
Factor	*p*-Value	B	95% CI	*p*-Value
**EORTC QLQ-C30**				
Global QOL	0.037	0.129	−0.136–0.394	0.332
Physical functioning	0.158	0.015	−0.493–0.522	0.954
Role functioning	0.780	0.153	−0.313–0.619	0.512
Emotional functioning	0.010	0.183	−0.163–0.529	0.291
Cognitive functioning	0.010	0.645	0.131–1.158	0.015
Social functioning	0.193	−0.205	−0.563–0.153	0.255
**HADS**				
Anxiety subscale	0.037	1.043	−1.865–2.341	0.821
Depression subscale	0.010	1.262	−3.247–1.841	0.581
**Patient and disease factors**				
ASA (ref)				
ASA II	0.062	−0.503	−17.087–16.082	0.952
ASA III	0.062	−9.513	−28.544–9.518	0.319
Age	0.620	0.156	−0.310–0.622	0.503
Sex	0.744	−5.930	−14.419–2.558	0.166
Colon vs. rectal	0.761	−2.526	−15.776–10.725	0.703
Neoadjuvant chemotherapy	0.647	−0.303	−25.950–25.344	0.981
Neoadjuvant radiotherapy	0.824	−4.495	−26.400–17.411	0.681
Stage (ref)				
Stage 2	0.741	−7.308	−21.127–6.511	0.292
Stage 3	0.352	−4.257	−16.146–7.632	0.474
Stage 4	0.491	−2.684	−23.703–18.335	0.798
**Surgical factors**				
MIS vs. open	0.131	−5.540	−17.498–6.419	0.356
Stoma vs. no stoma	0.196	−9.573	−23.903–4.757	0.185
Type of surgery (ref)				
Subtotal/total colectomy	0.203	−16.834	−40.707–7.040	0.162
Anterior resection	0.762	0.727	−11.300–12.754	0.904
Low anterior resection	0.012	39.060	14.861–63.260	0.002
Abdominoperineal resection	0.125	3.677	−17.074–24.427	0.723
Hartmanns surgery	0.431	22.017	−8.275–52.308	0.150
**Surgical outcomes**				
Readmission	0.066	0.096	−16.539–16.731	0.991
CCI score	0.517	0.194	−0.537–0.245	0.454

EORTC QLQ C-30—European Organization for the Research and Treatment of Cancer Quality of Life Questionnaire; QOL—Quality of life; HADS—Hospital anxiety and depression scale; ASA—American Society of Anesthesiologist; MIS—minimally invasive surgery; CCI—comprehensive complication index.

**Table 3:** Association between preoperative QLQ-C30/HADS mean scores and postoperative outcomes.

	**Postoperative Complications**					
	**Univariate Analysis**			**Multivariate Analysis (R^2^: 0.151, χ** **^2^** **: 9.194, *p* = 0.306)**		
	**No**	**Yes**	***p*-Value**	**OR**	**95% CI**	***p*-Value**
**EORTC QLQ-C30**						
Global QOL	72.16	64.46	0.081	0.985	0.955–1.015	0.315
Physical functioning	93.94	91.37	0.401	0.991	0.940–1.045	0.747
Role functioning	95.08	92.16	0.379	1.007	0.961–1.054	0.779
Emotional functioning	83.90	83.09	0.983	0.993	0.956–1.032	0.730
Cognitive functioning	96.21	93.14	0.388	0.958	0.902–1.017	0.159
Social functioning	91.67	86.74	0.241	0.984	0.948–1.021	0.394
**HADS**						
Anxiety subscale	4.77	4.71	0.545	0.816	0.650–1.024	0.079
Depression subscale	2.51	3.41	0.235	1.203	0.913–1.584	0.189
	**Comprehensive complication index score (CCI)**					
	**Univariate analysis**			**Multivariate analysis (R^2^: 0.134, F: 1.320, *p* = 0.249)**		
	**Beta**		***p*-value**	**OR**	**95% CI**	***p*-value**
**EORTC QLQ-C30**						
Global QOL	−0.074		0.517	0.047	−0.147–0.240	0.632
Physical functioning	−0.277		**0.014**	−0.296	−0.656–0.065	0.106
Role functioning	−0.189		0.098	0.031	−0.278–0.341	0.842
Emotional functioning	−0.051		0.660	−0.001	−0.259–0.258	0.997
Cognitive functioning	−0.182		0.111	−0.134	−0.522–0.254	0.492
Social functioning	−0.190		0.095	−0.106	−0.357–0.145	0.403
**HADS**						
Anxiety subscale	0.084		0.470	−0.739	−2.156–0.677	0.301
Depression subscale	0.196		0.088	1.410	−0.350–3.170	0.114
	**30-day readmission**					
	**Univariate analysis**			**Multivariate analysis (R^2^: 0.322, χ** **^2^** **: 13.129, *p* < 0.001)**		
	**No**	**Yes**	***p*-value**	**OR**	**95% CI**	***p*-value**
**EORTC QLQ-C30**						
Global QOL	68.57	70.83	0.980	1.021	0.964–1.082	0.482
Physical functioning	92.95	91.67	0.233	1.044	0.921–1.183	0.501
Role functioning	93.81	93.75	0.755	1.036	0.953–1.127	0.402
Emotional functioning	84.29	77.08	0.765	1.037	0.955–1.126	0.385
Cognitive functioning	91.43	72.92	**0.005**	0.928	0.841–1.024	0.139
Social functioning	91.43	72.92	**0.004**	0.925	0.867–0.988	**0.019**
**HADS**						
Anxiety subscale	4.49	6.88	0.252	0.978	0.670–1.426	0.906
Depression subscale	2.84	3.50	0.305	1.122	0.707–1.781	0.626
	**Length of hospitalization**					
	**Univariate analysis**			**Multivariate analysis (R^2^: 0.156, F: 1.577, *p* = 0.148)**		
	**Beta**		***p*-value**	**OR**	**95% CI**	***p*-value**
**EORTC QLQ-C30**						
Global QOL	−0.049		0.667	0.080	−0.163 −0.323	0.513
Physical functioning	−0.331		**0.003**	−0.620	−1.073–−0.167	**0.008**
Role functioning	−0.115		0.316	0.107	−0.282–0.497	0.584
Emotional functioning	−0.013		0.909	0.057	−0.269–0.383	0.727
Cognitive functioning	−0.101		0.379	0.001	−0.488–0.488	0.999
Social functioning	−0.040		0.731	0.064	−0.252–0.380	0.689
**HADS**						
Anxiety subscale	0.086		0.459	−0.323	−2.105–1.460	0.719
Depression subscale	−0.185		0.107	1.857	−0.358–4.072	0.099

EORTC QLQ C-30—European Organization for the Research and Treatment of Cancer Quality of Life Questionnaire; QOL—Quality of life; HADS—Hospital anxiety and depression scale.

## Data Availability

The data presented in this study are available on request from the corresponding author.
